# *DHCR7* mutations linked to higher vitamin D status allowed early human migration to Northern latitudes

**DOI:** 10.1186/1471-2148-13-144

**Published:** 2013-07-09

**Authors:** Valerie Kuan, Adrian R Martineau, Chris J Griffiths, Elina Hyppönen, Robert Walton

**Affiliations:** 1 Queen Mary University of London, Barts and The London School of Medicine and Dentistry, Blizard Institute, 58 Turner Street, London E1 2AB, UK; 2 MRC Centre of Epidemiology for Child Health and Centre for Paediatric Epidemiology and Biostatistics, UCL Institute of Child Health, 30 Guildford Street, London WC1N 1EH, UK; 3 School of Population Health, University of South Australia, Adelaide, Australia

**Keywords:** Evolutionary selection, Vitamin D, *DHCR7*, Fixation index, Long range haplotype test

## Abstract

**Background:**

Vitamin D is essential for a wide range of physiological processes including immune function and calcium homeostasis. Recent investigations have identified candidate genes which are strongly linked to concentrations of 25-hydroxyvitamin D. Since there is insufficient UVB radiation to induce year-round cutaneous synthesis of vitamin D at latitudes distant from the equator it is likely that these genes were subject to forces of natural selection. We used the fixation index (F_ST_) to measure differences in allele frequencies in 993 individuals from ten populations to identify the presence of evolutionary selection in genes in the vitamin D pathway. We then explored the length of haplotypes in chromosomes to confirm recent positive selection.

**Results:**

We find evidence of positive selection for *DHCR7,* which governs availability of 7-dehydrocholesterol for conversion to vitamin D_3_ by the action of sunlight on the skin. We show that extended haplotypes related to vitamin D status are highly prevalent at Northern latitudes (Europe 0.72, Northeast Asia 0.41). The common *DHCR7* haplotype underwent a recent selective sweep in Northeast Asia, with relative extended haplotype homozygosity of 5.03 (99th percentile). In contrast, *CYP2R1*, which 25-hydroxylates vitamin D, is under balancing selection and we found no evidence of recent selection pressure on *GC*, which is responsible for vitamin D transport.

**Conclusions:**

Our results suggest that genetic variation in *DHCR7* is the major adaptation affecting vitamin D metabolism in recent evolutionary history which helped early humans to avoid severe vitamin D deficiency and enabled them to inhabit areas further from the equator.

## Background

Vitamin D is essential in calcium homeostasis and bone health, with deficiency causing rickets and osteomalacia. In recent decades, vitamin D deficiency has also been implicated in the pathogenesis of other conditions, including cancers, autoimmune diseases, infections and cardiovascular diseases [1].

**Figure 1:**
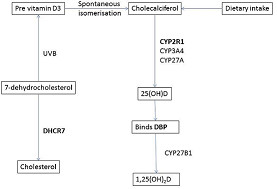
**Metabolic pathways involving genes linked to 25-hydroxyvitamin D levels.***DHCR7* encodes 7-dehydrocholesterol reductase, which converts 7-dehydrocholesterol to cholesterol, thereby reducing availability for vitamin D synthesis in the skin. *CYP2R1* encodes 25-hydroxylase, which converts Vitamin D to 25(OH)D. *GC* encodes Vitamin D Binding Protein (DBP) which is a glycosylated alpha-globulin that transports vitamin D metabolites from the gut and skin to target end-organs. Proteins linked to 25-hydroxyvitamin D levels are indicated in bold.

The main source of vitamin D is the ultraviolet irradiation of 7-dehydrocholesterol in the skin, which produces vitamin D_3_ (cholecalciferol). 7-dehydrocholesterol is thus a precursor both for vitamin D_3_ and for cholesterol – formation of the latter being catalysed by DHRC7 (Figure 1). Vitamin D can also be obtained from the diet, primarily from oily fish, although dietary sources are usually inadequate for normal requirements. Vitamin D from the skin and dietary sources is converted to 25-hydroxyvitamin D (25[OH]D), which is the major circulating vitamin D metabolite and generally used as a measure of vitamin D status [1]. This reaction is catalysed by microsomal (CYP2R1, CYP3A4) and mitochondrial (CYP27A) 25-hydroxylases. 25(OH)D is bound to vitamin D binding protein (GC) in the circulation. Genetic variants of GC differ with respect to their affinity for 25(OH)D. This, in turn, influences the availability of 25(OH)D to cells which convert it to 1,25-dihydroxyvitamin D (1,25[OH]_2_D) which is the biologically active metabolite [2].

Exposure to UVB in sunlight is the prime determinant of serum 25(OH)D concentration, but this effect is modified by biological factors such as skin pigmentation [3] and age-related reductions in cutaneous synthesis [4].

Twin and family studies suggest that heritability is responsible for a significant degree of variability in 25(OH)D levels [5,6], and recent genome-wide association studies have identified specific genes associated with 25(OH)D concentrations [7-10]. Single nucleotide polymorphisms (SNPs) in or near three genetic loci are strongly associated with 25(OH)D levels: *GC*[7,8,10], on Chromosome 4, which encodes Vitamin D binding protein; *DHCR7*[7,8], on Chromosome 11, which encodes 7-dehydrocholesterol reductase; and *CYP2R1*[7,8], on Chromosome 11, which encodes vitamin D 25-hydroxylase.

Given the pleiotropic functions of vitamin D in maintaining human health, and the established evidence for genetic influence on circulating 25(OH)D levels, we looked for evidence that vitamin D status has played a part in the adaptive evolution of humans. To this end, we searched for signals of natural selection in these three genes strongly associated with 25(OH)D levels.

Strategies for finding signatures of selection include: a) identification of a high frequency of function-altering mutations; b) a reduction in genetic diversity; c) high-frequency derived alleles; d) allele frequency differences between populations; e) unusually long haplotypes [11,12]. We focused on the latter two strategies, as these detect selective events in more recent times, and in specific populations. Thus we aimed to track the period in evolutionary history when early humans spread from Africa to northern latitudes where sunlight is more limited and cutaneous synthesis of vitamin D is very much reduced or absent in winter months [1].

Firstly, we investigated whether any of the markers associated with circulating 25(OH)D levels had outlying fixation index (F_ST_) values conditional on their expected heterozygosity (*H*_*e*_). F_ST_ is a commonly used statistic for measuring population differentiation; such differences in allele frequency among human populations will have accumulated after the major migrations out of Africa 50,000 to 75,000 years ago [12]. We ranked the pairwise-F_ST_ values of individual SNPs associated with vitamin D status against those of all SNPs on the remainder of the chromosome upon which the gene was located, thereby using these remaining alleles to generate an empirical distribution of chromosome-wide F_ST_ values. This allowed us to assess the evidence for positive selection at these loci, whilst avoiding confounding by population demographic history [13].

Next, we looked for the presence of extended haplotypes in these loci, comparing haplotype length with all core haplotypes of similar frequency on each chromosome. We evaluated the length of homozygosity of these haplotypes across extended chromosomal distances. Finally, we compared the extent of homozygosity of these haplotypes with others of similar frequency, in order to verify that the haplotypes had risen rapidly to high frequency before recombination events had time to disrupt linkage disequilibrium.

## Results

### Genetic markers near *DHRC7* show signals of positive selection and marked differences in allele frequencies between populations

We used the HapMap3 (International Haplotype Map Project, Phase 3, release 2) dataset to examine 15 of the 18 SNPs significantly associated with circulating 25(OH)D levels in recent meta-analyses conducted in populations of European descent [7]. Three of the SNPs, rs17467825, rs4945008 and rs10741657 were not in the HapMap3 dataset. The mean 25(OH)D concentrations by genotype for each SNP in the 1958 British Birth Cohort are shown in Additional file 1: Table S1.

Three SNPs near *DHCR7-* rs12785878, rs7944926 and rs3794060 - were identified as candidates for positive selection, and three near *CYP2R1* - rs2060793, rs1993116 and rs7116978 - for balancing selection using a neutrality test (Table 1). We analysed the allele frequencies of these SNPs in ten Hapmap3 populations: Africans in Southwest USA (ASW); Utah residents with ancestry from Northern and Western Europe (CEU); Chinese in Denver, Colorado (CHB); Gujerati in Houston, Texas (GIH); Japanese in Tokyo and Han Chinese in Beijing (JPT + CHB); Luhya in Kenya (LWK); Mexicans in Los Angeles (MEX); Maasai in Kenya (MKK); Toscans in Italy (TSI); Yoruba in Nigeria (YRI) (Tables 2 and 3).

**Table 1:** **Sample *H***_***e ***_**and F**_**ST **_**values at 5 *GC*, 5 *DHCR7 *and 5 *CYP2R1 *SNPs calculated in LOSITAN**

**Loci**	**H**_**e**_	**F**_**ST**_	P*
***DHCR7 SNPs***			
rs3794060	0.463422	0.265612	**0.997845**
rs7944926	0.467318	0.25473	**0.996788**
rs12785878	0.467134	0.254663	**0.99678**
rs12800438	0.491453	0.162411	0.922132
rs4944957	0.502397	0.127569	0.794576
***CYP2R1 SNPs***			
rs7116978	0.457296	0.007108	**0.004184**
rs2060793	0.468166	0.000742	**0.001499**
rs1993116	0.44101	0.021711	**0.024486**
rs12794714	0.434566	0.109713	0.674206
rs10500804	0.435266	0.110771	0.681743
***GC SNPs***			
rs2282679	0.306487	0.075355	0.451885
rs3755967	0.310383	0.068546	0.395229
rs1155563	0.331944	0.107236	0.671098
rs2298850	0.280963	0.094884	0.590703
rs7041	0.445035	0.142463	0.858668

*P (Simulated F_ST_ < sample F_ST_). The outlier loci above the 0.975 and below the 0.025 quantiles are shown in bold, and indicate positive and balancing selection, respectively.

**Table 2:** Allele frequencies of DHCR7 SNPs identified as having undergone positive selection in ten Hapmap populations

**SNP**	**Allele**	**Allele frequencies**									
		**ASW**	**CEU**	**CHD**	**GIH**	**JPT + CHB**	**LWK**	**MEX**	**MKK**	**TSI**	**YRI**
rs3794060	C	0.711	0.274	0.587	0.832	0.589	0.941	0.612	0.923	0.225	0.884
	T	0.289	0.726	0.413	0.168	0.411	0.059	0.388	0.077	0.775	0.116
rs7944926	A	0.684	0.277	0.587	0.832	0.584	0.941	0.612	0.92	0.223	0.844
	G	0.316	0.723	0.413	0.168	0.416	0.059	0.388	0.08	0.777	0.156
rs12785878	G	0.684	0.274	0.587	0.832	0.586	0.941	0.612	0.92	0.225	0.844
	T	0.316	0.726	0.413	0.168	0.414	0.059	0.388	0.08	0.775	0.156

**Table 3:** Allele frequencies of CYP2R1 SNPs identified as having undergone balancing selection in ten Hapmap populations

**SNP**	**Allele**	**Allele frequencies**									
		**ASW**	**CEU**	**CHD**	**GIH**	**JPT + CHB**	**LWK**	**MEX**	**MKK**	**TSI**	**YRI**
rs7116978	T	0.395	0.363	0.352	0.411	0.349	0.359	0.397	0.23	0.297	0.333
	C	0.605	0.637	0.648	0.589	0.651	0.641	0.603	0.77	0.703	0.667
rs2060793	A	0.412	0.394	0.352	0.431	0.347	0.367	0.414	0.305	0.343	0.342
	G	0.588	0.606	0.648	0.569	0.653	0.633	0.586	0.695	0.657	0.658
rs1993116	A	0.289	0.398	0.347	0.426	0.348	0.2	0.397	0.212	0.338	0.245
	G	0.711	0.602	0.653	0.574	0.652	0.8	0.603	0.788	0.662	0.755

The distribution of allele frequencies was similar within each population for the three polymorphisms near *DHCR7* reflecting the strong linkage disequilibrium in this region of the human genome. Strikingly, the major and minor alleles were reversed for all three SNPs in the Europeans (CEU and TSI) when compared to other populations. Large differences in allele frequencies between populations may signal a locus that has undergone positive selection in one geographical area.

An empirical distribution of F_ST_ values for all the SNPs on chromosome 11 in the HapMap3 dataset was constructed. The values were ranked from highest to lowest. Pairwise F_ST_ values for the *DHCR7* SNPs rs12785878, rs7944926 and rs3794060 between the European and African, and the European and Gujarati populations were above the 95th percentile relative to 70,973 SNPs on Chromosome 11. F_ST_ values were also above the 95th percentile for ASW vs LWK and ASW vs MKK at rs7944729 (Tables 4 and 5). Such high F_ST_ values can be generated when the direction or strength of selection differs among populations.

**Table 4:** **Pairwise F**_**ST **_**values for rs1279187 and rs7944926**

	**GIH**	**LWK**	**MKK**	**YRI**
**ASW**	not significant	0.20 (95.8th)	0.20 (97.3rd)	not significant
**CEU**	0.47 (98.1st)	0.61 (99.5th)	0.62 (99.9th)	0.48 (97.2nd)
**TSI**	0.52 (99.9th)	0.66 (99.7th)	0.67 (99.9th)	0.53 (98.9th)

**Table 5:** **Pairwise F**_**ST **_**values for rs3794060**

	**GIH**	**LWK**	**MKK**	**YRI**
**ASW**	not significant	0.17 (95.6th)	0.18 (97.1st)	not significant
**CEU**	0.47 (98.1st)	0.61 (99.5th)	0.63 (99.9th)	0.53 (98.6th)
**TSI**	0.52 (99.9th)	0.66 (99.7th)	0.68 (99.9th)	0.58 (99.2nd)

### Haplotype analysis suggests that *DHCR7* markers linked to high vitamin D status have undergone positive selection in Northeast Asia

**Figure 2:**
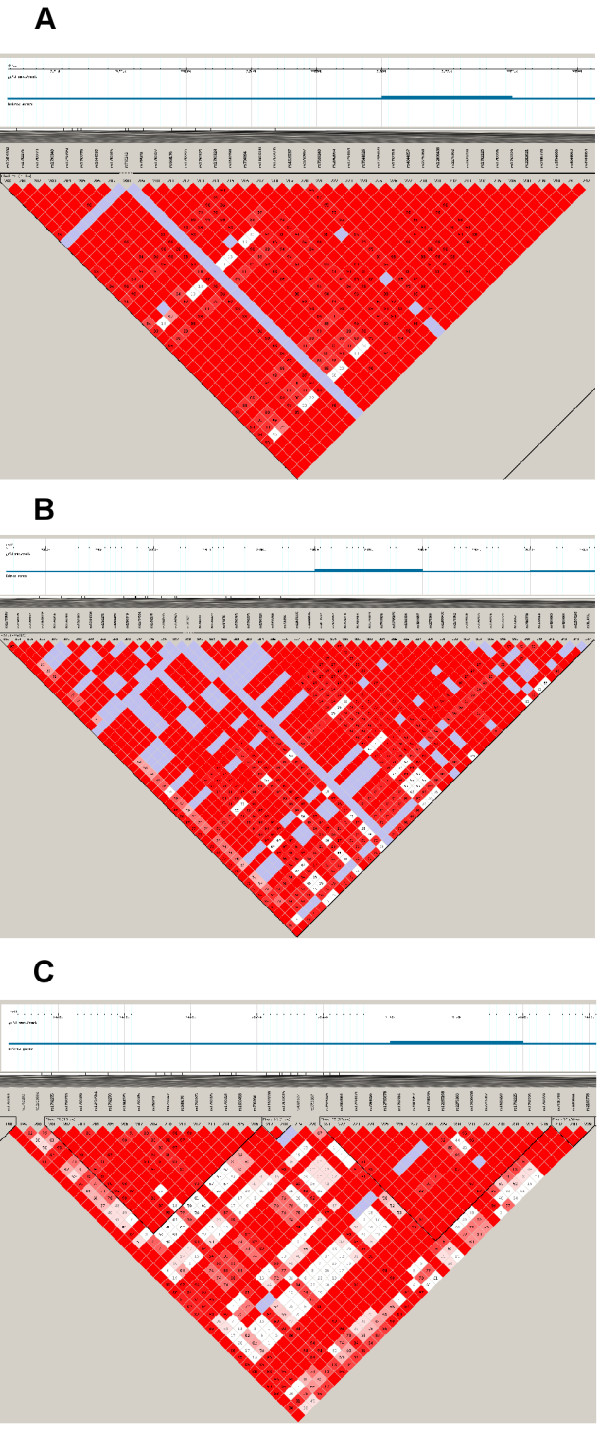
**Linkage disequilibrium of SNPs in and around DHCR7 and NADSYN1 for A) JPT + CHB, B) CEU and C) YRI.** The numbers within each square indicate the D’ values. Squares are colour-coded as follows: white: D’ < 1, LOD < 2; blue: D’ = 1, LOD < 2; pink: D’ < 1, LOD ≥ 2; and bright red: D’ = 1, LOD ≥ 2.

Another signal of a recent selective sweep is a long chromosomal region with strong linkage disequilibrium around the selected site. This large haplotype block was observed in the European, Northeast Asian, Mexican and Gujarati populations (Figure 2). The block, which varied between 63 kb and 102 kb, depending on the specific population, included all five SNPs associated with vitamin D status near *DHCR7* (rs7944926, rs12785878, rs12800438, rs4944957 and rs3794060). This was in contrast to the African populations, which had more haplotype blocks that were shorter, and no block contained all five SNPs (Figure 2). This observation indicates that the underlying haplotype structures in this region and the evolutionary forces driving these structures are different between the African and other populations, with the rate of recombination (compared to genetic drift) being higher in Africans.

A signature of positive selection is indicated by unusually long haplotypes and high population frequency [14]. Long range haplotypes persist for relatively short periods of time, since, after 30,000 years random recombination events tend to break down the haplotype, leaving fragments that are too short to detect [12]. We therefore next employed a long range haplotype test – Extended Haplotype Homozygosity (EHH), to assess whether these alleles had risen in prevalence so rapidly that there had been insufficient time for recombination to break down linkage disequilibrium with alleles at neighbouring loci [14].

A core region was defined, comprising 17 adjacent SNPs in *DHCR7* and a neighbouring gene *NADSYN1* (rs7944926, rs12785878, rs1792316, rs4944957, rs12280295, rs12285168, rs2276360, rs12800438, rs2276362, rs1629220, rs1792225, rs1792226, rs1792229, rs7131218, rs2282621, rs2186778 and rs3794060). This core region included the five SNPs associated with circulating 25(OH)D levels, in addition to non-synonymous coding variants in *NADSYN1*. In none of the African populations – ASW, LWK, MKK or YRI – could a haplotype block comprising these seventeen SNPs be formed, reinforcing the suggestion of relatively high effects of recombination in these populations. For the other six populations, the 17 SNPs defined four core haplotypes denoted *DHCR7-CH1* to *4* for core haplotypes 1 to 4 (Table 6).

**Table 6:** Core haplotype frequencies in six populations

**Core haplotype**	**Core alleles**	**Core haplotype frequencies in six populations**					
		**CEU**	**CHD**	**GIH**	**JPT + CHB**	**MEX**	**TSI**
**DHCR7-CH1**	GTCGTGCAGCCCACTCT	0.717	0.406	0.165	0.406	0.4	0.75
**DHCR7-CH2**	AGCATGGGATCTGCCCC	0.212	0.388	0.472	0.406	0.29	0.205
**DHCR7-CH3**	AGAATGGGACTCACCTC	0	0.141	0.352	0.109	0.3	0.028
**DHCR7-CH4**	AGCATGGGACCCACTCC	0	0.047	0	0.074	0	0

Core alleles refer to those of the seventeen SNPs in order from left to right: rs7944926, rs12785878, rs1792316, rs4944957, rs12280295, rs12285168, rs2276360, rs12800438, rs2276362, rs1629220, rs1792225, rs1792226, rs1792229, rs7131218, rs2282621, rs2186778, rs3794060.

**Figure 3:**
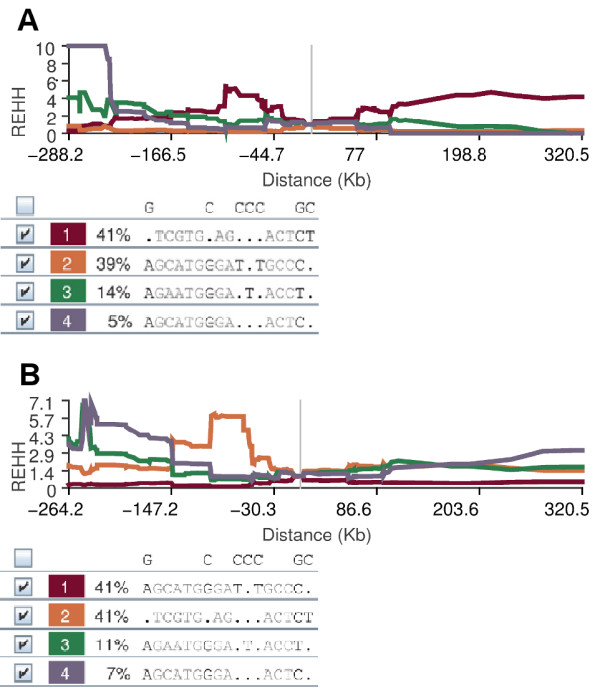
**Long range haplotype analysis of *DHCR7/NADSYN1 *core haplotypes.** REHH versus genomic distance plots of DHCR7-CH1 to CH4 for **A)** CHD and **B)** JPT + CHB are shown.

For each core haplotype the relative EHH (REHH) was calculated. REHH is the factor by which EHH decays on the core haplotype compared with the decay of EHH on all the other core haplotypes combined. Haplotypes were placed in 20 groups based on their frequency, at 5% intervals. The significance of the REHH values was assessed by comparing their percentiles within these 5% frequency groups for all possible core haplotypes on Chromosome 11. The REHH value at 0.25 cM for *DHCR7-CH1* was 4.90 (99th percentile of core haplotypes within 5% of its frequency) for CHD, and 5.03 (99th percentile of core haplotypes within 5% of its frequency) for JPT + CHB (Figure 3).

### Genetic variants in vitamin D binding protein (*GC*) and 25 hydroxylase (*CYP2R1*) show no evidence of recent evolutionary selection

F_ST_ analysis for SNPs near *CYP2R1* showed F_ST_ values below the 2.5% quantile compared to all other SNPs on the chromosome, an effect which could be produced by balancing selection. However, long-range haplotype analysis found no evidence for recent selective sweeps in any HapMap3 population for the core haplotypes containing the SNPs associated with vitamin D status. None of the SNPs in *GC* associated with circulating 25(OH)D levels showed evidence of selection in either F_ST_ or long range haplotype analysis.

## Discussion

We searched for evidence of positive evolutionary selection in genetic variants strongly linked to higher circulating 25(OH)D levels in recent, large-scale genome-wide association studies [7-10]. We now show for the first time unique signatures of recent positive selection at the *DHCR7* genetic locus. The same haplotype carrying these polymorphisms has risen to very high frequencies in both Europe (0.72) and in Northeast Asia (0.41). DHCR7 converts 7-dehydrocholesterol, which is a precursor for Vitamin D, into cholesterol. Thus reduced activity variants of DHRC7 would increase the availability of 7-dehydrocholesterol for cutaneous vitamin D synthesis. This implies that *DHCR7* mutations associated with higher vitamin D status conferred a survival advantage which allowed early humans to avoid severe deficiency when migrating to northern latitudes.

Rare loss-of-function mutations in *DHCR7* have been previously described and are associated with Smith-Lemli-Opitz syndrome (SLOS), in which affected homozygous individuals have greatly elevated serum 7-dehydrocholesterol levels and correspondingly low serum cholesterol [15]. It has previously been suggested that these rare variants might confer a survival advantage to heterozygotes by increasing 7-dehydrocholesterol availability and hence vitamin D synthesis [15].

One suggested mechanism by which this might occur is reduction in cephalopelvic disproportion due to rickets which in the past was a common cause of obstructed labour [16]. Low vitamin D status is also associated with low sperm count in men and with disorders of ovulation in women [17]. In addition to these effects on reproductive fitness, mortality from infectious diseases, particularly those affecting children and young adults could be a powerful selective force. Examples of such diseases linked to low vitamin D status have been clearly demonstrated in archaeological specimens from *Homo erectus* engaged in northerly migration [18].

Here we show that highly prevalent polymorphisms with a much smaller effect on 25(OH)D levels than those causing SLOS may nevertheless have exerted a strong selective force in human evolution by similar mechanisms. Whilst lower cholesterol levels arising from reduced DHCR7 activity may be advantageous in reducing the risk of heart disease and stroke, these effects are seen in later life and hence less likely to be a selective force in evolution. Thus vitamin D status rather than cholesterol level is more likely to be a driving force for natural selection.

Despite the higher prevalence of alleles associated with increased 25(OH)D levels in Europeans compared to other populations including Northeast Asians, a detailed examination of *DHCR7* haplotype homozygosity found no evidence of a recent selective sweep in Europeans. However, REHH for the haplotype associated with higher 25(OH) D levels was significant when compared to other SNPs of similar frequency on chromosome 11 in the Northeast Asian populations. The significant pairwise F_ST_ values between European and non-European populations suggest that there was an evolutionary drive in European populations for an allele which increased circulating 25(OH)D levels more than 70,000 years ago, but that the mutation did not achieve fixation. Although pairwise F_ST_ values between Northeast Asian and other populations were not significant, the finding of significant REHH values in Northeast Asian populations suggest that a selective event subsequently occurred approximately 30–50,000 years ago.

This suggests that mutations associated with higher vitamin D status were positively selected at higher latitudes in both continents over different periods due to a variety of environmental pressures, as they proffered an evolutionary advantage. These genetic variants then rose quickly to high frequencies in humans living in areas distant from the equator.

The differences in timing of the selective events between the European and Northeast Asian populations may in part reflect the diets of the respective populations in the late Pleistocene and early Holocene. The inhabitants of Europe during this period are thought to have had a diet that was rich in mammal and fish meat, which have a high vitamin D content [19-21]. In East Asia, in contrast, plant foods may have become increasingly important in the human diet with intensive exploitation of certain types of flora [22,23]. The comparatively vitamin D-rich diet of the European inhabitants at this time may be the reason the *DHCR7* mutation did not reach fixation, while the low vitamin D content in the diet of the East Asians may have added to the selection pressure on *DHCR7* over this period.

7-dehydrocholesterol is found in high concentrations in the epidermis [24]. Chen, et al. showed that the conversion of epidermal 7-dehydrocholesterol in hypopigmented skin is much more efficient than in highly pigmented skin. This suggests that sufficient previtamin D_3_ can be synthesized in Caucasians but not in people with heavily pigmented skin after a brief exposure to summer noon sunlight even at high latitudes [25]. Dependency upon sunlight for vitamin D synthesis may also explain why 7-dehydrocholesterol levels are three to eight times higher in non-feathered skin areas of birds such as the legs and feet when compared to body skin which is covered with feathers [26].

These studies show that humans and animals have adapted mechanisms to exploit optimal methods of synthesiszing vitamin D in response to their environment. The increased frequencies of *DHCR7* alleles associated with higher vitamin D status in the hypopigmented populations of Europe and Northeast Asia may represent yet another adaptation which conferred a survival advantage allowing early humans to avoid severe deficiency when migrating to northern latitudes.

Ethnic differences in vitamin D status have long been recognised [27-30], although the evolutionary factors responsible for the biological differences have not been elucidated. *SLC24A5*, a gene associated with skin pigmentation, shows clear evidence of adaptive selection in those of European ancestry but interestingly is not associated with vitamin D status in recent genome wide association studies [31,32]. This highly unexpected finding may suggest that factors other than vitamin D status are driving natural selection on the basis of skin colour. However vitamin D genome-wide analyses to date have been conducted mainly in European populations and thus the full range of *SLC24A5* genotypes may not have been fully explored.

The three genes that we studied contribute a relatively modest proportion of the variation in circulating 25(OH)D levels and of these only *DHCR7* shows marked differences in allele frequencies across populations. Whilst these small differences in vitamin D status appear to be sufficient to drive processes of natural selection, it seems likely that other important genetic mechanisms are still to be discovered, some of which may involve interactions between genes. Environmental factors such as sun exposure, latitude and diet will clearly play an important part and may also interact with genetic influences on vitamin D status [33].

With the exception of rs7041 in *GC*, the SNPs identified in the genome-wide association studies as being associated with vitamin D status are found in non-coding regions of the genome. These regions may exercise regulatory functions on relevant genes or alternatively the polymorphisms identified may be in linkage disequilibrium with neighbouring functional polymorphisms [34,35]. Further studies are required to determine how these polymorphisms effected the association with vitamin D status.

Because of the strong linkage disequilibrium in this genomic region it is possible that the positive selection that we detected relates not to the *DHCR7* gene, but to *NADSYN1*, which is situated close to *DHCR7* in the genome and was included in the core haplotype. *NADSYN1* encodes NAD synthetase, an enzyme that catalyzes the final step in the biosynthesis of nicotinamide adenine dinucleotide (NAD) from nicotinic acid adenine dinucleotide (NaAD). NAD is an important cofactor in redox reactions and is also involved in post-translational modification of proteins; however there is no known biological connection between this gene and vitamin D metabolism.

It is notable that whilst the major alleles for SNPs in *GC* and *DHCR7* are associated with higher 25(OH)D levels in Europeans, the opposite was true for four of the SNPs near *CYP2R1* (Additional file 1: Table S1). This makes the suggestion of balancing selection acting near the *CYP2R1* locus plausible, although the biological reasons for this are unclear.

Neither *GC* nor *CYP2R1* showed evidence of positive selection in our analysis. One explanation for this may be that selection in the *GC* or *CYP2R1* genes took place earlier than that in the *DHCR7* gene, and hence was not detected by the methods used in this study. Alternatively, it may be the level of vitamin D_3_ rather than 25(OH)D that influences survival and brings about evolutionary selection. If this were the case, then the availability of 7-dehydrocholesterol to promote synthesis of vitamin D in the skin would be more important than 25 hydroxylase activity or the contribution of D binding protein (Figure 1). Whilst this conclusion would be contrary to most current opinion there is some evidence that 25-hydroxylation may occur in a range of target tissues [36,37].

## Conclusions

This observation of positive evolutionary selection highlights the physiological importance of vitamin D in humans and sheds new light on the crucial role of DHCR7 in the synthetic pathway. The finding that the same molecular variant is selected in both northern Europe and Northeast Asia is highly unusual and strengthens the argument that individuals with low DHCR7 activity had a survival advantage at northern latitudes despite variations in other environmental factors in different continents. The unique signal of evolutionary selection, amongst all the genetic variants linked to higher 25(OH)D levels, points to the primacy of DHCR7 amongst the vitamin D synthetic enzymes and will help to direct future research towards a hitherto neglected area of vitamin D metabolism.

## Methods

### Genotype data

Phased genotype data were obtained from release 2 of the HapMap 3 dataset [38]. Only paired chromosomes were studied. These were from 993 individuals: 53 of African ancestry in Southwest USA (ASW); 113 from Utah with Northern and Western European ancestry from the Centre d’Etude du Polymorphisme Humain (CEPH) collection (CEU); 85 from the Chinese population in Metropolitan Denver, Colorado (CHB); 88 from the Gujerati population in Houston, Texas (GIH); 170 from the Japanese population in Tokyo and the Han Chinese population in Beijing (JPT + CHB); 90 from the Luhya population in Webuye, Kenya (LWK); 50 of Mexican ancestry in Los Angeles (MEX); 143 from the Maasai population in Kinyawa, Kenya (MKK); 88 from the Tuscan population in Italy (TSI); and 113 from the Yoruba population in Ibadan, Nigeria (YRI).

### F_ST_ vs. heterozygosity

An F_ST_ -outlier detection approach [39] using the LOSITAN [40] software package was implemented to identify SNPs which may have undergone positive or balancing selection. The expected distribution of Wright’s inbreeding coefficient, F_ST_, against the expected heterozygosity, *H*_*e*_, was constructed under an island model of migration with neutral markers. This was used to identify outlier loci with excessively high or low F_ST_ compared to neutral expectations, which would be candidates for selection. 10,000 simulation replicates were used (infinite allele model) for a sample size of 993 individuals.

### Pairwise F_ST_

Pairwise F_ST_ values to assess population differentiation were calculated as described by Weir and Cockerham with Nei’s correction for sample size [41-43] between the ten populations. Values were ranked from highest to lowest, with those above the 95th percentile regarded as significant.

### Haplotype block definition

Haplotype blocks were constructed using the LD-based empirical block definition proposed by Gabriel, et al. [44]. Haploview 3.0 [45] was used to estimate and view the detailed blocks and their underlying haplotype structure.

### EHH and REHH

The EHH statistic uses the decay of linkage disequilibrium to estimate the age of the haplotypes. It is defined as the probability that any two randomly chosen chromosomes carrying a particular core haplotype have the same extended haplotype from the core region to a distance x. The relative EHH (REHH), which compares the EHH of a core haplotype to that of other core haplotypes at the same locus, corrects for local variation in recombination rates [14]. EHH and REHH values were calculated for core regions which contained the SNPs of interest.

The significance of REHH was tested using empirical data from the HapMap3 dataset of the entire chromosome on which the core region was situated. REHH values were calculated for core haplotypes in all haplotype blocks for the relelvant chromosome. The haplotypes were then placed into 20 bins based on their frequency. Analysis was carried out using Sweep software (Varilly P, Fry B, and Sabeti P, http://www.broadinstitute.org/mpg/sweep/).

### 1958 Birth cohort

Serum 25(OH)D concentrations were measured using an automated IDS OCTEIA enzyme-linked immunosorbent assay (ELISA) (Dade-Behring BEP2000 analyzer), standardized according to the mean from Vitamin D External Quality Assessment Scheme (DEQAS) [46]. Genetic data for the cohort were obtained from by Affymetrix 6.0 and Illumina 550 K Infinium methods from two genome wide sub-studies (Wellcome Trust Case–control Consortium 2 and Type 1 Diabetes Genetics Collaboration) [47,48].

## Abbreviations

ASW: African ancestry in Southwest USA; CEU: Utah residents with Northern and Western European ancestry from the CEPH collection; CHD: Chinese in Metropolitan Denver, Colorado; cM: centiMorgan; EHH: Extended haplotype homozygosity; GIH: Gujarati Indians in Houston, Texas; GWAS: Genome wide association study; JPT + CHB: Japanese in Tokyo, Japan + Han Chinese in Beijing, China (MERGED); LWK: Luhya in Webuye, Kenya; MEX: Mexican ancestry in Los Angeles, California; MKK: Maasai in Kinyawa, Kenya; REHH: Relative extended haplotype homozygosity; SNP: Single nucleotide polymorphism; TSI: Toscans in Italy; YRI: Yoruba in Ibadan, Nigeria (West Africa).

## Competing interests

The authors declare that they have no competing interests.

## Authors’ contributions

RW, ARM, VK and CJG contributed to study design. VK and EH took part in data analysis. VK, RW, ARM, EH and CJG interpreted the results. VK, RW and ARM drafted the manuscript. All authors read and approved the final manuscript.

## Supplementary Material

Additional file 1: Table S1 Geometric mean of 25-hydroxyvitamin D by SNP genotypes in the 1958 British Birth Cohort (*n* = 5,233). Click here for file
