# Supplementary material for: DHCR7 mutations linked to higher vitamin D status allowed early human migration to Northern latitudes
Source: BMC Evol Biol. 2013 Jul 9;13:144. doi: 10.1186/1471-2148-13-144 (PMC3708787; doi:10.1186/1471-2148-13-144)
Supplement: Additional file 1: Table S1 — Geometric mean of 25-hydroxyvitamin D by SNP genotypes in the 1958 British Birth Cohort (n = 5,233). [file 1471-2148-13-144-S1.doc]

|  | 25-Hydroxyvitamin D geometric mean (95% CI), nmol/L | | | | | |
| --- | --- | --- | --- | --- | --- | --- |
| ***GC*** | | | | | | |
| **SNPs** (Major > Minor allele) | **rs2282679** (T>G) | **rs3755967** (C>T) | **rs17467825** (A>G) | **rs1155563** (T>C) | **rs2298850** (G>C) | **rs7041** (C>A) |
| Major homozygotes | 54.2 (53.3, 55.3) | 54.3 (53.3, 55.3) | 54.3 (53.3, 55.3) | 53.8 (52.8, 54.9) | 54.3 (53.3, 55.3) | 54.5 (53.2, 55.8) |
| Heterozygotes | 50.3 (49.3, 51.3) | 50.3 (49.3, 51.3) | 50.3 (49.3, 51.3) | 50.4 (49.4, 51.5) | 50.2 (49.2, 51.3) | 51.5 (50.5, 52.4) |
| Minor homozygotes | 46.7 (44.7, 48.7) | 46.8 (44.8, 48.8) | 46.8 (44.9, 48.9) | 46.1 (44.0, 48.3) | 46.7 (44.6, 48.8) | 48.5 (47.1, 50.0) |
| ***DHCR7/NADSYN1*** | | | | | | |
| **SNPs** (Major > Minor allele) | **rs12785878** (T>G) | **rs7944926** (G>A) | **rs12800438** (A>G) | **rs3794060** (T>C) | **rs4945008** (G>A) | **rs4944957** (G>A) |
| Major homozygotes | 52.8 (52.0, 53.7) | 52.8 (52.0, 53.7) | 52.8 (52.0, 53.7) | 52.8 (52.0, 53.7) | 52.8 (52.0, 53.7) | 52.8 (52.0, 53.7) |
| Heterozygotes | 50.4 (49.3, 51.6) | 50.3 (49.2, 51.5) | 50.4 (49.3, 51.5) | 50.3 (49.2, 51.5) | 50.5 (49.4, 51.6) | 50.5 (49.4, 51.6) |
| Minor homozygotes | 50.5 (47.3, 54.0) | 50.5 (47.3, 54.0) | 50.7 (47.5, 54.1) | 50.5 (47.3, 53.8) | 50.4 (47.2, 53.7) | 50.5 (47.3, 54.0) |
| ***CYP2R1*** | | | | | | |
| **SNPs**(Major > Minor allele) | **rs10741657** (G>A) | **rs2060793** (G>A) | **rs1993116** (G>A) | **rs12794714** (G>A) | **rs10500804** (T>A) | **rs7116978** (C>T) |
| Major homozygotes | 50.1 (49.1, 51.2) | 50.3 (49.2, 51.3) | 50.1 (49.1, 51.2) | 52.7 (51.4, 53.9) | 52.7 (51.4, 53.9) | 50.6 (49.6, 51.6) |
| Heterozygotes | 52.6 (51.6, 53.6) | 52.5 (51.5, 53.5) | 52.6 (51.6, 53.6) | 52.5 (51.6, 53.5) | 52.5 (51.6, 53.5) | 52.6 (51.6, 53.6) |
| Minor homozygotes | 53.5 (51.6, 55.4) | 53.4 (51.6, 55.3) | 53.4 (51.5, 55.2) | 49.1 (47.7, 50.5) | 49.1 (47.7, 50.5) | 53.1 (51.2, 55.2) |
